# Overview of preventive practices provided by primary care physicians: A cross-sectional study in Switzerland and France

**DOI:** 10.1371/journal.pone.0184032

**Published:** 2017-09-05

**Authors:** Paul Sebo, Hubert Maisonneuve, Bernard Cerutti, Jean-Pascal Fournier, Nicolas Senn, Cédric Rat, Dagmar M. Haller

**Affiliations:** 1 Primary Care Unit, Faculty of Medicine, University of Geneva, Geneva, Switzerland; 2 Unit of Research and Development in Medical Education, Faculty of Medicine, University of Geneva, Geneva, Switzerland; 3 Department of General Practice, Faculty of Medicine, University of Nantes, Nantes, France; 4 Department of Ambulatory Care and Community Medicine, University of Lausanne, Lausanne, Switzerland; 5 French National Institute of Health and Medical Research, Unit 1232-team 2, Nantes, France; 6 Department of Community, Primary Care and Emergency Medicine & Department of Paediatrics, Geneva University Hospitals, Geneva, Switzerland; University of Zurich, SWITZERLAND

## Abstract

**Background:**

A range of preventive practices are recommended to reduce the burden of chronic diseases. The aim of our study was to describe the preventive practices of French-speaking primary care physicians.

**Methods:**

A cross-sectional survey was conducted in 2015 in a randomly selected sample of 1100 primary care physicians (700 in Switzerland, 400 in France). The physicians were asked how often they performed the following recommended preventive practices: blood pressure, weight and height measurements, screening for dyslipidemia, screening for alcohol use and brief intervention, screening for smoking (and brief advice for smokers), colon and prostate cancer screening, and influenza immunization. Response options on the five points Likert scale were never, rarely, sometimes, often, always. The physicians were considered to be performing the preventive practice regularly if they declared performing it often or always.

**Results:**

518 participants (47%) returned the questionnaire. The most commonly reported preventive practices were: blood pressure measurement (99%), screening for smoking (95%) and brief advice for smokers (95%). The least frequently reported practices were annual influenza immunization for at-risk patients <65 years (37%), height measurement (53%), screening for excessive alcohol use (60%) and brief advice for at-risk drinkers (67%). All other practices were reported by 70 to 90% of participants.

**Conclusion:**

Whereas some preventive practices now appear to be part of primary care routine, others were not applied by a large proportion of primary care physicians in our study. Further studies should explore whether these findings are related to miss-knowledge of common guidelines, or other implementation barriers in this primary care context.

## Introduction

Prevention is an essential part of primary care. Preventive practices are particularly important for the management of major modifiable risk factors, such as smoking, hypertension and dyslipidemia. [1] The United States Preventive Services Task Force (USPSTF) recommendations and the Canadian Task Force on Preventive Health Care (CTFPHC) offer evidence-based guidelines for the delivery of preventive care by primary care physicians (PCPs). [2,3] Physicians in French-speaking parts of Europe often rely on these North American guidelines, alongside country specific recommendations. For example, in Switzerland, recommendations were developed as part of EviPrev, [4] a national program which includes evidence-based recommendations drawn from Swiss and international guidelines. [2,3,5–8] In France, there are currently no global recommendations in relation to preventive care. Preventive care recommendations can be found in the French medical authorities’ guidelines for the management of specific disorders. [9,10]

It is essential to monitor primary care medicine, including preventive practices, to understand how the system operates and to achieve a high quality of care. [11] Despite the existence of evidence-based recommendations, the actual rates of preventive care in the US remain low: overall, only about half of recommended preventives services are provided. [9,11–13] The following reasons for lack of adherence have been reported: lack of time during the consultation, insufficient financial compensation for preventive care, lack of awareness of and absence of agreement between the various recommendations. [9]

In Continental Europe, most primary care studies to date examined preventive care in relation to specific disorders. For example, a systematic review revealed that approximately fifty studies regarding prevention activities in primary care were conducted in Switzerland between 1990 and 2010. [14] A large number of these studies showed that preventive care was effective in reducing the burden of chronic diseases. [14] However, with the exception of Collet’s study (see below), [15] the adherence to standard guidelines was examined only for specific conditions, such as type 2 diabetes, [16] cholesterol [17] and aspirin use for the primary prevention of coronary heart disease. [18] Collet et al. assessed the quality of a broad range of primary and secondary preventive care practices performed by PCPs. [15] They selected 37 indicators among those developed in a similar US study, [11,19] and showed that, overall, patients were exposed to 69% of the recommended preventive care practices. Prevention of cardiovascular risk factors was common (83%) whereas cancer screening rates were relatively low (<40%). However, the study was retrospective, limited to academic primary care settings, somewhat outdated (2005–2006), and the data were abstracted from medical charts. A recent work also identified an important lack of data regarding the quality of preventive care in Switzerland. [20]

In France, the literature on this topic is scarce. A large cross-sectional multicenter study was carried out in 2011–2012. Among 19’003 primary care consultations carried out by 128 PCPs, 0% to 78% focused on prevention (median 15%) showing therefore a large disparity among PCPs. [21] The study also showed that the proportion of consultations focusing on prevention was lower for PCPs with younger patients, more home visits or shorter consultations. Another study found that “systematic health examination and prevention” was the most common motive for encounter reported in primary care (19% of patients), the two following being “rhino-pharyngitis” (13%) and high blood pressure (12%). [22]

In order to add much needed data coming from Continental Europe to the literature on preventive practices in primary care, our aim was to report preventive care in various French-speaking areas: Western Switzerland (cantons of Geneva and Vaud) and two French regions (Alsace, Pays de la Loire). We sought to explore these practices in two countries in which prevention recommendations are issued in a different form, in order to develop hypotheses about the preferred way to implement preventive practices in these countries. Our aim was also to identify possible generational differences (age and professional experience) in these practices.

## Methods

### Participants

A random sample of 700 community-based PCPs practicing in Switzerland (canton of Geneva and Vaud) and 400 in France (Alsace and Pays de la Loire) was selected from a sampling frame consisting of all PCP members of the regional professional organization of physicians. The PCPs were invited to participate in the study per post. Reminder messages (maximum twice per PCP) were sent. All community-based PCPs were eligible for the study, except those who exclusively practiced complementary and alternative medicine. The initial sample originally also included 2300 randomly selected PCPs invited to participate by email. As response rates were extremely low (11%), we chose to exclude this group of PCPs from the study. The recruitment process and details about PCPs’ selection have been described elsewhere. [23]

### Sample size estimation

To be able to provide a 95% confidence interval no wider than +/- 4% for every estimate of prevalence of preventive practices, with an expected prevalence of about 50%, the minimal required sample size was 600. [9,11–13,19] Assuming a participation rate between 50 and 60%, 1100 PCPs had to be contacted. [24]

### Data collection

In Switzerland, each randomly selected PCP was contacted per post by a research assistant located in Geneva. In France, selected PCPs were contacted by the local professional associations: Union Régionale des Professionnels de Santé Alsace and Pays de la Loire. The PCPs were informed about the aim of the study and the procedure for completing and sending back the questionnaire.

### Development of the questionnaire

The questionnaire (see S1 Appendix pour the French version and S2 Appendix pour the English version of the questionnaire) included socio-demographic questions (age group, gender, number of half-days worked per week and number of working years in private practice) and questions about twelve preventive practices (see Box 1) assessed with a five point Likert scale ranging from “never performed” (1) to “always performed” (5). PCPs were asked to focus on preventive practices for asymptomatic adults without any risk factors. The screening frequencies listed in Box 1 were chosen according to most common recommendations (see below).

**Box 1. List of the twelve preventive measures explored in this study**

- Blood pressure measurement (at least once per year)
- Weight measurement (at least once)
- Height measurement (at least once)
- Screening for dyslipidemia
- Screening for at-risk drinking (at least once)
- Advice to decrease drinking for at-risk drinkers
- Screening for smoking (at least once)
- Advice to stop smoking for smokers (at least once per year)
- Screening for colorectal cancer
- No widespread screening for prostate cancer
- Annual influenza immunization for patients ≥65 years
- Annual influenza immunization for at-risk patients <65 years

The selection of the twelve preventive practice indicators was based on a consensus within the research team. The following indicators had also been selected by previous authors: blood pressure, weight and height measurements, screening for excessive alcohol use and brief intervention, screening for smoking (and brief advice for smokers), colorectal cancer screening, influenza immunization for patients ≥ 65 years and at-risk patients <65 years) [15] Cholesterol measurement was added to this list because it is highly recommended. [2,3,8] Finally, refraining from systematic prostate cancer screening was also added to the list because several Swiss and French medical agencies (Swiss Society of Urology, Swiss Academy of Medical Sciences, Swiss Medical Board, Haute Autorité de Santé) recently recommended against systematic screening. [4,6–8,25–27]

Type 2 diabetes screening was considered as targeted screening (limited to populations with particular risks such as obesity or relevant family history) and therefore was not selected for our project. [5,6] Screening for breast and cervical cancers were not selected either, because in some locations these screening activities are performed by gynecologists, or through public health screening programs.

Additional questions were asked for two preventive practices, in order to assess whether they were performed according to common recommendations. For prostate cancer, we assessed whether screening was proposed only in the context of a shared and informed decision making process between PCPs and patients. PCPs were considered to be performing this preventive practice if they stated that they never or rarely screened or that they screened with shared decision regardless of the frequency with which they screened. For influenza immunization, in addition to patients ≥65 years, we asked whether PCPs offered it systematically to 1) patients <65 years at risk, defined as: patients with chronic heart, lung, liver and kidney diseases, splenic dysfunction, immune deficiency, living in nursing home), 2) patients having regular contact with at-risk patients or with infants <6 months, and 3) caregivers. [28,29]

A pretest was carried out among seven PCPs to ensure that the questionnaire was understandable and easy to complete. The questionnaires did not contain any identification data about respondents and all collected data remained confidential throughout the study.

### Consent and ethical approval

Implicit consent was assumed when a PCP returned a completed questionnaire. We did not collect any data about the PCPs who declined participation. In Switzerland, informed consent waiving was granted by the Research Ethics Committee of Geneva (approval by the Ethics Committee is not necessary under Swiss law for studies in which no personal health-related data are collected). In France, the research protocol was approved by the *groupe nantais d’éthique dans le domaine de la santé* (ref: 2015-09-06).

### Statistical analyses

For each item, except prostate cancer, we computed the proportion of PCPs delivering each recommendation, defined as the proportion of PCPs reporting that they performed the measure often or always. For prostate cancer, adhesion to recommendations (not to screen…) was defined as the proportion of PCPs stating that they never or rarely screened, or that they screened with shared decision regardless of how frequently they did so. We investigated whether preventive practices were associated with PCPs’ characteristics (country, gender and age group), using frequency tables and chi-square tests. We also investigated the link between the number of recommended measures provided by the PCPs and several cofactors (age, gender, location of the practice, number of half-days worked per week, number of working years in private practice), using analyses of variance. All the available covariates were included in the multivariate analysis of variance model. Then a backward stepwise elimination procedure was used so as to remove any covariates associated with a p-value higher than 0.05. All analyses were undertaken with TIBCO Spotfire S+® 8.1 for Windows (TIBCO Software Corporation, Palo Alto, CA, USA) or R version 3.2.2 (R Foundation for Statistical Computing, Vienna, Austria).

## Results

Overall, 518 participants returned the questionnaire (response rate: 47%). Table 1 presents their socio-demographic characteristics in both countries. The PCPs’ profile was relatively similar in the two countries regarding their gender (men: 61% in Switzerland vs. 66% in France). However, compared to their Swiss counterparts, French PCPs were slightly older (age ≥ 55 years: 57% vs. 49%), had been in private practice for longer (22 years vs. 17 years) and were working slightly more half-days per week (8.9 vs. 8.4 half-days per week).

**Table 1:** Primary care physicians’ characteristics in the two countries (n = 518).

Characteristics	Total	Switzerland	France	
	n^1^ (%)	n^1^ (%)	n^1^ (%)	p-value
Gender	*n = 509*	*n = 353*	*n = 156*	
male	318 (62.5)	215 (60.9)	103 (66.0)	0.3171
female	191 (37.5)	138 (39.1)	53 (34.0)	
Age group (years)	*n = 516*	*n = 354*	*n = 162*	
< 35	13 (2.5)	2 (0.6)	11 (6.8)	0.0002
35–44	104 (20.2)	79 (22.3)	25 (15.4)	
45–54	133 (25.8)	99 (28.0)	34 (21.0)	
55–64	207 (40.1)	130 (36.7)	77 (47.5)	
> 64	59 (11.4)	44 (12.4)	15 (9.3)	
	**mean ± SD**	**mean ± SD**	**mean ± SD**	
Number of half-days worked per week	8.6 ± 2.3	8.4 ± 2.4	8.9 ± 2.0	0.0181
Number of working years in private practice	18.7 ± 11.0	16.9 ± 10.5	22.4 ± 11.4	<0.0001

^1^ n = number with factor considered

The proportion of PCPs who reported that they regularly performed the preventive practices varied (Table 2). Overall, the most commonly reported practices were regular blood pressure measurement (99%), screening for smoking (95%), and advice to stop smoking for smokers (95%). The least often reported practices were annual influenza immunization for at-risk patients <65 years (37%), height measurement (53%), screening for at-risk drinking (60%) and advice to decrease drinking for at-risk drinkers (67%). Three preventive practices were significantly more frequently reported by PCPs in Switzerland (height measurement; screening for at-risk drinking; and refraining from systematic screening for prostate cancer). The least commonly reported practices were, in Switzerland, annual influenza immunization for at-risk patients <65 years (36%) and, in France, height measurement (40%) and annual influenza immunization for at-risk patients <65 years (41%).

**Table 2:** Proportion of primary care physicians delivering the twelve recommended measures of preventive care, stratified by country.

Characteristics	Total (n = 518)		Switzerland (n = 355)		France (n = 163)		
	%	95% CI %	%	95% CI %	%	95% CI %	p-value
Blood pressure measurement (at least once per year)	**98.5**	97.4–99.5	**98.0**	96.6–99.5	**99.4**	98.2–100	0.4350
Weight measurement (at least once)	**88.2**	85.4–91.0	**87.9**	84.5–91.3	**89.0**	81.1–93.8	0.1449
Height measurement (at least once)	**53.3**	49.0–57.6	**59.4**	54.3–64.5	**39.9**	32.4–47.4	0.0001
Screening for dyslipidemia	**75.7**	72.0–79.4	**77.7**	73.4–82.1	**71.2**	64.2–78.1	0.1308
Screening for at-risk drinking (at least once)	**60.0**	55.8–64.3	**66.2**	61.3–71.1	**46.6**	39.0–54.3	<0.0001
Advice to decrease drinking for at-risk drinkers	**66.6**	62.5–70.7	**67.3**	62.4–72.2	**65.0**	57.7–72.4	0.6792
Screening for smoking (at least once)	**95.0**	93.1–96.9	**96.3**	94.4–98.3	**92.0**	87.9–96.2	0.0613
Advice to stop smoking for smokers	**95.2**	93.3–97.0	**94.9**	92.6–97.2	**95.7**	92.6–98.8	0.8714
Screening for colorectal cancer	**83.0**	79.8–86.2	**85.1**	81.4–88.8	**78.5**	72.2–84.8	0.0863
Screening for prostate cancer	**76.8**	73.2–80.5	**83.7**	79.8–87.5	**62.0**	54.5–69.4	<0.0001
Annual influenza vaccine for patients ≥ 65 years	**89.6**	86.9–92.2	**89.6**	86.4–92.8	**89.6**	84.9–94.3	0.8789
Annual influenza vaccine for at-risk patients < 65 years	**37.3**	33.1–41.4	**35.5**	30.5–40.5	**41.1**	33.6–48.7	0.2590

Table 3 presents the proportion of PCPs delivering the twelve preventive care practices according to their gender and age group. Two recommendations were significantly more frequently reported by female PCPs compared to their male counterparts: screening for at-risk drinking and screening for smoking, whereas only one preventive practice (refraining from systematic screening for prostate cancer) was more frequently followed by PCPs <55 years compared to older PCPs.

**Table 3:** Proportion of primary care physicians delivering the twelve recommended measures of preventive care, stratified by gender and age group.

Characteristics	Men (n = 318)		Women (n = 191)			Age <55 (n = 250)		Age >55 (n = 266)		
	%	95% CI %	%	95% CI %	p-value	%	95% CI %	%	95% CI %	p-value
Blood pressure measurement (at least once per year)	**98.4**	97.1–99.8	**98.4**	96.7–100	0.7139	**98.8**	97.5–100	**98.1**	96.5–99.8	0.7886
Weight measurement (at least once)	**85.8**	82.0–89.7	**91.6**	87.7–95.6	0.0717	**87.6**	83.5–91.7	**88.7**	84.9–92.5	0.7964
Height measurement (at least once)	**51.9**	46.4–57.4	**55.5**	48.4–62.5	0.4847	**53.6**	47.4–59.8	**53.0**	47.0–59.0	0.9629
Screening for dyslipidemia	**76.7**	72.1–81,4	**74.3**	68.2–80.5	0.6161	**76.0**	70.7–81.3	**75.2**	70.0–80.4	0.9108
Screening for at-risk drinking (at least once)	**54.4**	48.9–59.9	**70.7**	64.2–77.1	0.0004	**62,4**	56.4–68.4	**57.9**	52.0–63.8	0.3399
Advice to decrease drinking for at-risk drinkers	**67.0**	61.8–72.1	**66.5**	59.8–73.2	0.9871	**70.4**	64.7–76.1	**63.5**	57.7–69.3	0.1182
Screening for smoking (at least once)	**93.4**	90.7–96.1	**97.9**	95.9–99.9	0.0387	**96.4**	94.1–98.7	**94.0**	91.1–96.8	0.2838
Advice to stop smoking for smokers	**94.3**	91,8–96.9	**96.3**	93.7–99.0	0.4255	**94.8**	92.0–97.6	**95.5**	93.0–98.0	0.8737
Screening for colorectal cancer	**83.6**	79.6–87.7	**81.7**	76.2–87.2	0.6522	**84.4**	79.9–88.9	**82.0**	77.3–86.6	0.5328
Screening for prostate cancer	**75.8**	71.1–80.5	**80.6**	75.0–86.2	0.2465	**83.2**	78.6–87.8	**71.4**	66.0–76.9	0.0021
Annual influenza vaccine for patients ≥ 65 years	**90.9**	87.7–94.0	**88.5**	84.0–93.0	0.4714	**91.6**	88.2–95.0	**88.0**	84.1–91.9	0.2254
Annual influenza vaccine for at-risk patients < 65 years	**38.7**	33.3–44.0	**35.6**	28.8–42.4	0.5487	**39.6**	33.5–45.7	**35.3**	29.6–41.1	0.3634

Finally, Table 4 presents the number of recommended practices reported by the participating PCPs, according to their socio-demographic characteristics. Overall, they reported performing approximately nine preventive care practices. The multivariate analysis of variance showed that female PCPs, those working more than eight half-days per week, and those practicing in Switzerland tended to perform more measures of preventive care than the others.

**Table 4:** Number of recommended measures of preventive care provided by primary care physicians, according to socio-demographic characteristics of the responders.

Characteristics				Multivariate		
	Mean ± SD	p value ^1^	All twelve measures %	adjusted p value ^1^	Difference number of measures	95% CI
Gender						
male	**9.1 ± 1.9**	0.1159	7.6	**0.0636**		
female	**9.4 ± 1.8**		6.5		**+0.48**	**+0.12 +0.83**
Age group (years)						
< 35	**8.6 ± 1.9**	0.0133	0.0	0.1816		
35–44	**9.7 ± 1.6**		10.9		§	§
45–54	**9.2 ± 1.9**		7.9		§	§
55–64	**9.1 ± 1.9**		5.2		§	§
> 64	**8.8 ± 1.9**		7.3		§	§
Number of half days worked per week						
≤ 8	**9.1 ± 1.9**	0.5440	7.8	**0.0082**		
> 8	**9.3 ± 1.8**		6.7		**+0.46**	**+0.11 +0.81**
Number of working years in private practice						
≤ 18	**9.4 ± 1.9**	0.0251	9.0	0.2287		
> 18	**9.0 ± 1.8**		5.4		§	§
Location of the practice						
Switzerland	**9.4 ± 1.8**	<0.0001	8.9	**<0.0001**		
France	**8.7 ± 1.9**		3.4		**-0.87**	**-0.52–1.23**

§ not selected in the adjusted multivariate model

^1^ Difference between the subgroups (Fisher F tests following the analysis of variance)

## Discussion

We showed that PCPs had screening practices in accordance with most common recommendations for prevention, though certain measures were less often performed (above all, annual influenza immunization for at-risk patients <65 years). We also showed that PCPs regularly carried out approximately nine out of twelve measures of preventive care, with female PCPs, those working more than eight half-days per week and those practicing in Switzerland tending to report slightly more preventive care practices than the others.

Our study compares favorably with the Swiss study conducted ten years ago by Collet et al in academic primary care settings. [15] The most important differences are seen with screening for colorectal cancer (83% of PCPs had practices in accordance with common recommendations in the current study vs. 35% in Collet’s study) and annual influenza immunization for patients ≥65 years (90% vs. 35%), screening for smoking (95% vs. 79%) and advice to stop smoking for smokers (95% vs. 72%). Three measures were performed slightly more often by PCPs practicing in academic primary care settings than by those involved in our study: weight (95% vs. 88%) and height measurement (75% vs. 53%), and advice to decrease drinking for at-risk drinkers (77% vs. 67%). Higher rates of influenza immunization for patients ≥65 years and screening for colorectal cancer in the present study may suggest a better acceptance with the years, as two older Swiss studies also found low rates of influenza immunization (51%), respectively of screening for colorectal cancer (25% for men and 17% for women) among community-based PCPs. [30,31] Another explanation may be that doctors naturally tend to overreport positive, socially desirable behaviors (social desirability bias). [32] The clinical significance of the comparison with Collet’s study is indeed limited by the fact that this latter study had a retrospective design, with data abstracted from medical charts a decade before our present study.

To our knowledge, only one study was performed on this topic in France: Gelly et al. selected 64 indicators considered as being related to preventive care among those developed in ICPC-2 (see abbreviations). [21] Using passive observers who assessed 19’003 medical consultations carried out by 128 PCPs, the authors showed that the proportion of consultations per PCP focusing on preventive care was very variable, ranging from 0% to 78% (median proportion: 15%); unfortunately, the comparison with our findings is impossible, as only aggregate scores were presented in the paper.

Although height measurements are relatively simple, we showed that they are much less often performed than weight measurement, and the difference is even greater for French PCPs. As height is needed to determine body-mass index (BMI), this finding could mean that many PCPs do not screen for obesity using BMI, despite the prominent position PCPs have in the identification and management of this condition. [33] It is not excluded that some PCPs screen for abdominal obesity using waist and/or hip circumference, or waist-to-hip ratio, but we recently showed that the great majority of PCPs do not use these new anthropometric measurements. [33]

The recommendations about prostate cancer screening are rather well followed by Swiss (84% only propose screening with shared decision making), less so by French PCPs (62%). Recommendations about prostate cancer screening have only recently been changed in view of new evidence showing little effects of screening on global and specific mortality. [8,26] Since some doubts remain, many countries recommend shared decision-making, including Switzerland and France. [8,26] Our finding suggests that PCPs (and maybe patients) may find it more difficult in France than in Switzerland to adopt the transition towards theses new recommendations. Alternatively, the difference in prostate cancer screening between the two countries could in part be explained by the fact that, compared to their Swiss counterparts, French PCPs were slightly older (and had been in private practice for longer). Indeed, several authors have shown that older PCPs tend to have lower adherence to recent guidelines. [34,35]

At-risk alcohol use is highly prevalent worldwide and is a leading preventable cause of death and disability. Screening for this risk behavior has been recommended for a long time, yet is relatively infrequently performed by our sample of PCPs (60% in the entire sample, 66% in Switzerland and 47% in France). [36] Studies from the US show even lower results: in a survey of internists and family physicians (n = 210), only one-third of patients were screened (except during the initial visit). [37] Another study revealed that less than one-third of 7371 adults who visited a PCP in the past year reported being asked about their alcohol or drug use. [38] Finally, according to a survey of young adults aged 18–39 (n = 3799), younger patients seem to be asked about alcohol use even less frequently (one-fourth in patients aged 18–20). [39] These findings could be explained in two ways. PCPs could face difficulties defining who is clearly at risk. [40] This could be particularly the case in countries in which alcohol use is considered as part of the “cultural heritage” and life style, and commonly regarded as a normal habit. [41] In addition, PCPs may fear to stigmatize their patients by defining that their alcohol use is excessive. In contrast, the large adherence (>90%) to recommendations to screen for smoking and advising to quit is reassuring. It also has the potential to contribute, in addition to national tobacco prevention programs and policy regulations, to the decline in smoking prevalence observed among adults in many high-income countries. [42,43]

It is interesting to note that the vast majority of PCPs provide annual influenza immunization to patients ≥65 years and to patients <65 years suffering from chronic heart or lung diseases, but not to other at-risk patients, despite the fact that they are also at high risk of complications in case of infection. In addition, immunization was infrequently proposed to regular contacts of at-risk patients and to infants <6 months, or patients <65 years living in nursing homes. These results suggest gaps in knowledge regarding the current guidelines or other reasons for not following these recommendations, such as false beliefs about the risk of infection in certain at-risk groups, lack of time and oversights. [44,45] Several factors could help to overcome these gaps, such as organization of national campaigns, implementation of mandatory immunization policies and use of recall systems. [44,45] Note that, in 2010–2011, the seasonal influenza vaccination coverage rate was 42.0% in Switzerland and 60.7% in France among patients 65 years and over, or with chronic diseases. [46,47]

We found that female (compared to male PCPs), those working more than eight half-days per week (compared to those working less), and those practicing in Switzerland (compared to those practicing in France), tend to follow more measures of preventive care. It has been shown that female and male doctors have different styles of care, female doctors tending to be more oriented than males toward prevention. [48,49] The finding that PCPs working more than eight half-days per week tend to follow more measures of preventive care can be explained by the fact that they may have more time available to discuss these issues with their patients than those working less.

Some of the reasons for not performing prevention practices (such as lack of time and method of reimbursement for medical care) may vary between countries, and thus may partly explain the country differences in our study. In France, the payment system is based on pre-fixed rates, whereas in Switzerland, it is based on fee-for-service which is related to the consultation time, thus allowing longer consultation times in this country. [21,50,51] The extent to which in France the absence of one unified preventive recommendations’ document also contributes to the lower uptake of preventive practices deserves further exploration.

Our study was not designed to describe the reasons for not performing prevention practices, but several hypotheses can be made. Several factors have been described in the literature (lack of time, gaps in knowledge, lack of mandatory postgraduate training and method of reimbursement for medical care) and were already mentioned above. The large number of guidelines, not only across but also within the countries (with different medical societies proposing different guidelines) can lead to conflicting recommendations and absence of agreement. In addition new knowledge leads to frequent updates of guidelines, which may be difficult for PCPs to follow in daily practice. Nation-wide campaigns and systematic performance monitoring could probably increase the proportion of PCPs conducting preventive practices in accordance with common recommendations. The aim of the SPAM project (Swiss Primary Health Care Active Monitoring), which was launched in Switzerland in 2010–2011 is to describe the performance and effectiveness of the Swiss primary care system and to identify potential avenues for improvement. [52] It explores three main domains: structure (accessibility, financing, workflow, and functioning of resources), output (medical training, management of knowledge, clinical/interpersonal care) and outcome (health status, patient/provider’s satisfaction and equity). The first results are expected soon. To our knowledge, there are no plans to launch a similar project in France.

### Limitations

Our study has several major limitations. Only PCPs practicing in Western Switzerland and two regions in France were invited to participate; this sample could not be representative of all PCPs practicing in French-speaking parts of Europe. Though the PCPs’ profile was relatively similar in the two countries, they were some differences (age distribution, mean number of days worked per week and experience as community-based PCP); these could play a role in the observed differences between the two countries. Our study was slightly underpowered, as only 518 PCPs agreed to participate whereas 600 were expected; however, this should have modest effects on our findings as we were able to detect statistically significant differences. The initial sample originally also included 2300 randomly selected PCPs invited to participate by email; as response rates were extremely low (11%), we chose to exclude this group of PCPs from the study, due to the high risk of introducing bias. We did not record PCPs’ mean length of consultation per patient and number of consultations per day; however, lack of time could explain why certain PCPs do not adhere to recommendations regarding preventive practices. No formal adjustments were made for multiple testing, given the mainly descriptive objective of the study. Caution should thus be taken when interpreting the significance of the reported associations. Finally, our findings could partially be explained by the fact that responders may overreport positive, socially desirable behaviors (social desirability bias); unfortunately, the extent of this bias cannot be assessed in our study, as we only recorded doctors’ preventive practices through auto-questionnaire, without « direct observation » of these practices.

In conclusion, though a majority of PCPs seem to have practices in accordance with most common recommendations for preventive care, our findings suggest that certain important measures are often not performed. Further studies should provide context-specific guidance about strategies to overcome the barriers to implementing primary care preventive care guidelines in French-speaking regions of Europe.

## Supporting information

S1 Appendix prevprim2a_plosone_appendix1. Questionnaire (French version).
(DOCX)

S2 Appendix prevprim2a_plosone_appendix2. Questionnaire (English version).
(DOCX)

## Abbreviations

PCPs: Primary care physicians
USPSTF: United States Preventive Services Task Force
CTFPHC: Canadian Task Force on Preventive Health Care
ICPC-2: International Classification of Primary Care, second version
BMI: body-mass index
SPAM project: Swiss Primary Health Care Active Monitoring
