# Supplementary material for: Overview of preventive practices provided by primary care physicians: A cross-sectional study in Switzerland and France
Source: PLoS One. 2017 Sep 5;12(9):e0184032. doi: 10.1371/journal.pone.0184032 (PMC5584957; doi:10.1371/journal.pone.0184032)
Supplement: S1 Appendix — Questionnaire (French version). (DOCX) [file pone.0184032.s001.docx]

***Evaluation des mesures de prévention primaire effectuées par les médecins de premier recours***

|  | 1. Effectuez-vous, chez les **adultes asymptomatiques**, les douze **mesures de prévention primaire** suivantes ?  ***Les patients présentant un risque augmenté de maladie*** *(comme par ex les patients obèses, hypertendus ou diabétiques, ou ceux ayant des ATCD personnels ou familiaux de cancer)* ***sont exclus*** | | Jamais | **Rarement** | **Parfois** | **Souvent** | **Toujours** | **Je ne sais pas** |
| --- | --- | --- | --- | --- | --- | --- | --- | --- |
| 1.1. Mesure de la **TAH systolique et diastolique** (au moins 1x/an) | | | ^1^ | ^2^ | ^3^ | ^4^ | ^5^ | ^6^ |
| 1.2. Mesure du **poids** (au moins 1x) | | | ^1^ | ^2^ | ^3^ | ^4^ | ^5^ | ^6^ |
| 1.3. Mesure de la **taille** (au moins 1x) | | | ^1^ | ^2^ | ^3^ | ^4^ | ^5^ | ^6^ |
| 1.4. Dépistage de l’**hypercholestérolémie** | | | ^1^ | ^2^ | ^3^ | ^4^ | ^5^ | ^6^ |
| a. A partir de quel âge le faites-vous généralement ? ____ ans  b. Jusqu’à quel âge le faites-vous généralement ? ____ ans  c. A quelle fréquence généralement ? Une fois tous les ____ ans  d. Faites-vous plutôt le cholestérol total ^1^ ou le bilan lipidique complet ^2^? | | |  |  |  |  |  |  |
| 1.5. Dépistage de la **consommation d’alcool à risque** (au moins 1x) | | | ^1^ | ^2^ | ^3^ | ^4^ | ^5^ | ^6^ |
| a. Utilisez-vous généralement un questionnaire validé ? ^1^ oui ^2^ non  b. Si oui, lequel ou lesquels ? ^1^ AUDIT ^2^ CAGE ^3^ MAST ^4^ autre | | |  |  |  |  |  |  |
| 1.6. Conseils de **diminuer** (si consommation d’alcool à risque) | | | ^1^ | ^2^ | ^3^ | ^4^ | ^5^ | ^6^ |
| a. Généralement pour les hommes buvant au moins ____ verres/sem.  b. Généralement pour les femmes buvant au moins ____ verres/sem.  c. Généralement pour les hommes buvant au moins ____ verres/occasion  d. Généralement pour les femmes buvant au moins ____ verres/occasion | | |  |  |  |  |  |  |
| 1.7. Dépistage du **tabagisme chronique** (au moins 1x) | | | ^1^ | ^2^ | ^3^ | ^4^ | ^5^ | ^6^ |
| 1.8. Conseils d’**arrêter de fumer** (au moins 1x/an) | | | ^1^ | ^2^ | ^3^ | ^4^ | ^5^ | ^6^ |
|  | | 1. Effectuez-vous, chez les **adultes asymptomatiques**, les douze **mesures de prévention primaire** suivantes (*suite…*) :  ***Les patients présentant un risque augmenté de maladie*** *(comme par ex les patients obèses, hypertendus ou diabétiques, ou ceux ayant des ATCD personnels ou familiaux de cancer)* ***sont exclus*** | Jamais | **Rarement** | **Parfois** | **Souvent** | **Toujours** | **Je ne sais pas** |
| 1.9. Dépistage du **cancer du côlon** | | | ^1^ | ^2^ | ^3^ | ^4^ | ^5^ | ^6^ |
| a. A partir de quel âge le faites-vous généralement ? ____ ans  b. Jusqu’à quel âge le faites-vous généralement ? ____ ans  c. Faites-vous le dépistage plutôt par coloscopie^1^ ou recherche de sang dans selles^2^  d. A quelle fréquence généralement ? Une fois tous les ____ ans | | |  |  |  |  |  |  |
| 1.10. Dépistage du **cancer de la prostate** par dosage du PSA | | | ^1^ | ^2^ | ^3^ | ^4^ | ^5^ | ^6^ |
| a. A partir de quel âge le faites-vous généralement ? ____ ans  b. Jusqu’à quel âge le faites-vous généralement ? ____ ans  c. A quelle fréquence généralement ? Une fois tous les ____ ans  d. Dans le cadre d’une « décision partagée » généralement ?  oui  non | | |  |  |  |  |  |  |
| 1.11. **Vaccination antigrippale** pour les ≥ 65 ans (1x/an) | | | ^1^ | ^2^ | ^3^ | ^4^ | ^5^ | ^6^ |
| 1.12. **Vaccination antigrippale** pour les < 65 ans à risque (1x/an) | | | ^1^ | ^2^ | ^3^ | ^4^ | ^5^ | ^6^ |
| a. C’est-à-dire (plusieurs réponses possibles) :  ^1^ maladie cardiaque chronique, ^2^ maladie pulmonaire chronique  ^3^ affection hépatique chronique, ^4^ insuffisance rénale chronique  ^5^ dysfonction splénique, ^6^ déficit immunitaire  ^7^ vit en EMS, ^8^ contact régulier avec l’une de ces 7 catégories  ^9^ contact régulier avec des nourrissons< 6mois, ^10^ personnel soignant | | |  |  |  |  |  |  |

Et pour finir, quelques questions sur vous-même…

| 2. Etes-vous... | ^1^ un homme | ^2^ une femme |
| --- | --- | --- |
| 3. Dans quelle tranche d’âge vous situez-vous ? | ^1^ 25-29 ans  ^2^ 30-34 ans  ^3^ 35-39 ans  ^4^ 40-44 ans  ^5^ 45-49 ans | ^6^ 50-54 ans  ^7^ 55-59 ans  ^8^ 60-64 ans  ^9^ ≥65 ans |
| 4. Combien de demi-jours travaillez-vous en moyenne par semaine (min 1, max 14) ? | demi-jours par semaine (min 1, max 14) | |
| 5. Quel est le code postal du cabinet ? |  | |
| 6. Depuis combien d’années êtes-vous installé(e) en pratique privée ? | ans | |

Nous vous remercions vivement d’avoir accepté de participer à cette enquête !
