# Supplementary material for: Overview of preventive practices provided by primary care physicians: A cross-sectional study in Switzerland and France
Source: PLoS One. 2017 Sep 5;12(9):e0184032. doi: 10.1371/journal.pone.0184032 (PMC5584957; doi:10.1371/journal.pone.0184032)
Supplement: S2 Appendix — Questionnaire (English version). (DOCX) [file pone.0184032.s002.docx]

***Overview of preventive practices provided by primary care physicians***

|  | 1. Do you perform these **twelve preventive practices** in **asymptomatic adults**?  ***Patients with increased risk of disease (****such as obese, hypertensive or diabetic patients, as well as patients with personal or family history of cancer)* ***are excluded*** | Never | **Rarely** | **Sometimes** | **Often** | **Always** | **I don’t know** |
| --- | --- | --- | --- | --- | --- | --- | --- |
| 1.1. **Systolic and diastolic blood pressure** measurement (at least once/year) | | ^1^ | ^2^ | ^3^ | ^4^ | ^5^ | ^6^ |
| 1.2. **Weight** measurement (at least once) | | ^1^ | ^2^ | ^3^ | ^4^ | ^5^ | ^6^ |
| 1.3. **Height** measurement (at least once) | | ^1^ | ^2^ | ^3^ | ^4^ | ^5^ | ^6^ |
| 1.4. Screening for **dyslipidemia** | | ^1^ | ^2^ | ^3^ | ^4^ | ^5^ | ^6^ |
| a. From what age do you generally perform this screening? ____ years  b. Until what age do you generally perform this screening? ____ years  c. At what frequency do you generally perform this screening? once every ____ years  d. Which diagnostic test do you generally use? total cholesterol ^1^ or full lipid profile ^2^ | |  |  |  |  |  |  |
| 1.5. Screening for **at-risk drinking** (at least once) | | ^1^ | ^2^ | ^3^ | ^4^ | ^5^ | ^6^ |
| a. Do you generally use a validated questionnaire to screen? ^1^ yes ^2^ no  b. If yes, which questionnaire(s)? ^1^ AUDIT ^2^ CAGE ^3^ MAST ^4^ other | |  |  |  |  |  |  |
| 1.6. Advice to **decrease drinking** (if at-risk drinking) | | ^1^ | ^2^ | ^3^ | ^4^ | ^5^ | ^6^ |
| a. Generally for men drinking at least ____ glasses per week  b. Generally for women drinking at least ____ glasses per week  c. Generally for men drinking at least ____ glasses per occasion  d. Generally for women drinking at least  ____ glasses per occasion | |  |  |  |  |  |  |
| 1.7. Screening for **smoking** (at least once) | | ^1^ | ^2^ | ^3^ | ^4^ | ^5^ | ^6^ |
| 1.8. Advice to **stop smoking** (at least once/year) | | ^1^ | ^2^ | ^3^ | ^4^ | ^5^ | ^6^ |
|  | 1. Do you perform these **twelve preventive practices** in **asymptomatic adults**?  ***Patients with increased risk of disease (****such as obese, hypertensive or diabetic patients, as well as patients with personal or family history of cancer)* ***are excluded*** | Never | **Rarely** | **Sometimes** | **Often** | **Always** | **I don’t know** |
| 1.9. Screening for **colon cancer** | | ^1^ | ^2^ | ^3^ | ^4^ | ^5^ | ^6^ |
| a. From what age do you generally perform this screening? ____ years  b. Until what age do you generally perform this screening? ____ years  c. Which diagnostic test do you generally use? colonoscopy ^1^ or stool-based test ^2^ ?  d. At what frequency generally? once every ____ years | |  |  |  |  |  |  |
| 1.10. Screening for **prostate cancer** using PSA | | ^1^ | ^2^ | ^3^ | ^4^ | ^5^ | ^6^ |
| a. From what age do you generally perform this screening? ____ years  b. Until what age do you generally perform this screening? ____ years  c. At what frequency generally? once every ____ years  d. Generally in the context of a shared decision process?  yes  no | |  |  |  |  |  |  |
| 1.11. **Influenza immunization** for patients ≥ 65 years (once/year) | | ^1^ | ^2^ | ^3^ | ^4^ | ^5^ | ^6^ |
| 1.12. **Influenza immunization** for at-risk patients < 65 years (once/year) | | ^1^ | ^2^ | ^3^ | ^4^ | ^5^ | ^6^ |
| (several possible answers) :  ^1^ chronic heart disease, ^2^ chronic lung disease  ^3^ chronic liver disease, ^4^ chronic kidney disease  ^5^ splenic dysfunction, ^6^ immune deficiency  ^7^ living in nursing home, ^8^ regular contact with one of these 7 categories  ^9^ regular contacts with infants< 6 months, ^10^ caregivers | |  |  |  |  |  |  |

Finally, a few questions about yourself…

| 2. Are you... | ^1^ a man | ^2^ a women |
| --- | --- | --- |
| 3. Which age group do you fall into? | ^1^ 25-29 years  ^2^ 30-34 years  ^3^ 35-39 years  ^4^ 40-44 years  ^5^ 45-49 years | ^6^ 50-54 years  ^7^ 55-59 years  ^8^ 60-64 years  ^9^ ≥65 years |
| 4. How many half-days do you generally work per week (min 1, max 14)? | half-days per week (min 1, max 14) | |
| 5. What is the postal code of your current practice? |  | |
| 6. How many years have you been in private practice? | years | |

We thank you very warmly for having agreed to participate in this survey!
